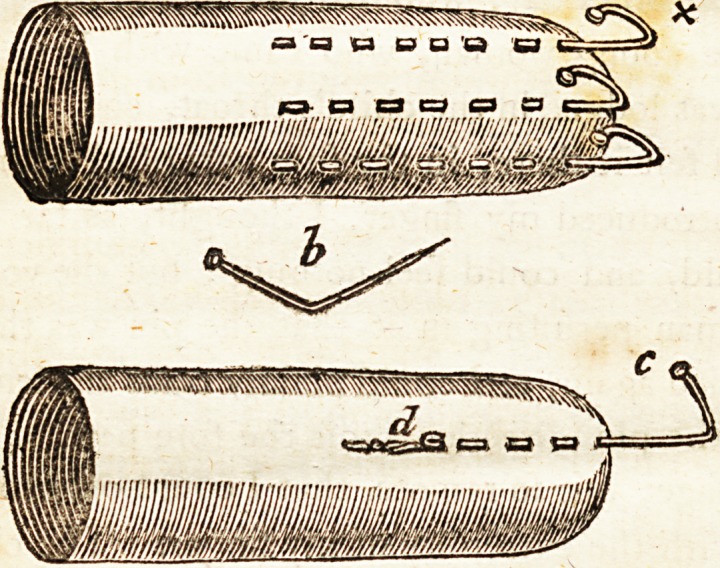# Case of a Child Who Swallowed a Pin

**Published:** 1785

**Authors:** 


VIII. *Caſe of a Child who ſwallowed a Pin. Communicated in a Letter to Dr.* Simmons, *F. R. S. by* William Boys, *Eſq. F. S. A. Member of the Corporation of Surgeons of London, and Surgeon, at Sandwich, in Kent.*

A Neighbour's child was brought to me the other day, who had juſt ſwallowed a pin. The young woman, who came with her, ſaid it was lodged in the child's throat, and that ſhe had felt it there, in the fore part of the throat. I introduced my finger, I thought, as far as I could, and could feel nothing: but the young woman perſiſting in it that the pin was there, I tried again, and could plainly trace the length of the pin directly acroſs the fore part of the paſſage, but ſo remote, that I could only touch it with the extremity of my finger. The child was continually making efforts to vomit, and was in great diſtreſs. It was therefore neceſſary to attempt to remove the pin as ſoon as poſſible.

I happened to have ſome very long lace pins, out of which I ſelected three, whoſe heads had no aſperities. Theſe I turned, with a pair of ſmall pliers, at their heads, ſo as to form hooks; taking care to turn the heads rather inwards, that there might be no hazard of their taking hold of the rugous parts of the oefophagus, and forming the angles in fuch manner, that if the hooks fhould get hold of the fhank of the pin, and the head of the pin fhould firft be loofened from its fituation, the angles might catch the head, and bring the pin away.

I ran thefe pins out and in feveral times through the fore finger of my buckfkin glove, and turned the points on the infide of the glove, fo that it feemed impoffible they fhould give way in the leaft. Upon introducing my finger thus armed into the child's throat, I was foiled by her ftruggles; but, on a fecond attempt, I found my finger entangled with fomething, and could not difengage it with a very confiderable effort. With a force, however, that that was frightful to uſe, I got my finger back again; but, to my great concern, without the pin. Blood followed immediately both from the mouth and noſe, and I concluded, at the inſtant, that the hooks, notwithſtanding my precautions, had lacerated the parts, and that I muſt have done conſiderable miſchief; but I was ſoon a good deal relieved from my anxiety by the child's ſaying ſhe was well, and that the pin was gone. I gave her a glaſs of water, which ſhe drank off without the leaſt difficulty, and went home. I deſired her mother to examine her ſtools for ſome days; and, the fourth day, ſhe found the pin, bent as repreſented at *b*, though it was ſtraight when ſwallowed.

I ſhould obſerve, that the hook, marked *x*, was drawn in the manner repreſented at *c*, and the point had torn its way through the glove, as at *d*; ſo great was the force neceſſary to diſengage the pin! It is clear that the head of the pin ſtood faſt; that the effort I made tore the point through the fleſh; that the point ſlipped from the hook; and that the child immediately ſwallowed the pin. No inconvenience has happened from the laceration. The child is eight years old.

In emergencies like theſe, one muſt act from the ſuggeſtion of the preſent moment. It would, perhaps, have been better if I could have introduced the hooks between the fore part of the oeſophagus and the pin, and ſo have raiſed the pin towards the wider part of the paſſage; but it lay ſo contiguous to the membrane, that, I think, this could not have been done.

*Sandwich,*

Sept. 11, 1785.

## Figures and Tables

**Figure f1:**