# Errata

**Published:** 1785

**Authors:** 


					p. 4.CI, 1. 33, for tbifc J turned, read theft J rent*
END OF V OL, VI. *

				

